# The Chronic Pain Myth Scale: Development and Validation of a French-Canadian Instrument Measuring Knowledge, Beliefs, and Attitudes of People in the Community towards Chronic Pain

**DOI:** 10.1155/2016/5940206

**Published:** 2016-09-25

**Authors:** Anaïs Lacasse, Judy-Ann Connelly, Manon Choinière

**Affiliations:** ^1^Département des Sciences de la Santé, Université du Québec en Abitibi-Témiscamingue, Rouyn-Noranda, QC, Canada; ^2^Département d'Anesthésiologie, Faculté de Médecine, Université de Montréal, Montréal, QC, Canada; ^3^Centre de Recherche du Centre Hospitalier de l'Université de Montréal (CRCHUM), Montréal, QC, Canada

## Abstract

*Background*. In order to better design awareness programs on chronic pain (CP), measurement of knowledge, beliefs, and attitudes of people in the community towards this condition is most useful.* Objectives*. To develop and validate a French-Canadian scale that could be used for this purpose.* Methods*. Items of the Chronic Pain Myth Scale (CPMS) were developed based on different information sources, reviewed by pain experts, and pretested. The CPMS was administered to 1555 participants among the general Quebec population.* Results*. The final CPMS contained 26 items allowing the calculation of three subscales scores (knowledge, beliefs, and attitudes towards people suffering from CP, biopsychosocial impacts of CP, and treatment of CP) which showed adequate internal consistency (*α* = 0.72–0.82). There were statistically significant differences in subscales scores between participants who reported suffering versus not suffering from CP, reported knowing versus not knowing someone who suffers from CP, and reported being versus not being a healthcare professional, which supports the construct validity of the scale.* Conclusions*. Our results provide preliminary evidence supporting the psychometric qualities of the use of the CPMS for the measurement of knowledge, beliefs, and attitudes towards CP among French-speaking individuals of the Quebec general population.

## 1. Introduction

Chronic pain (CP) affects approximately 11 to 29% of the adult Canadian population [1–5] and has major impacts on the physical functioning, the mental health, and the quality of life of those suffering from it [6–9]. It also constitutes an important economic burden as much for the patients as for the healthcare system and the different third-party payers [10, 11].

It is well documented that CP is commonly underreported, underrecognized, and underdiagnosed in clinical practice and in the community [12–16]. Its treatment remains suboptimal, mainly because of insufficient training of healthcare professionals, limited access to specialized pain clinics, suboptimal use or efficacy of treatment modalities, and lack of recognition of this condition [6, 13, 14, 16–18]. Heightened awareness coupled to better education about this condition for the healthcare professionals, the patients, and the general public is an important but a still neglected aspect in the efforts to improve CP management [17, 19]. Indeed, it is not rare to hear pain clinicians and researchers mentioning that negative beliefs and prejudices towards CP are still common in the community. In fact, several studies reported on stigmatization of patients suffering from CP and lack of empathy from the community, including healthcare professionals [20–22].

In order to better design and tailor awareness and education programs about CP, its impacts, and the ways to prevent/manage it, measurement of knowledge, beliefs, and attitudes of the general population towards this condition is most useful. However, available validated measurement scales have been originally designed for specific populations such as healthcare professionals [23–30] or patients suffering from different pain conditions [31–39], and none of them were intended for broader use in persons in the community irrespective of their background. In fact, scales designed to be used among healthcare professionals or patients sometimes asked about aspects that are specific to their biomedical expertise or condition (e.g., pain diagnosis, drug prescription, specific impacts of pain treatments, available scientific data, and how patients view or cope with their own symptoms). Although some of the available scales originally designed for patients have been used in pain-free subjects [35, 37, 40], some common misbeliefs towards people suffering from CP that are often heard in the community did not seem well covered. Other scales were validated in the general population, but only applicable to a specific type of CP (e.g., back pain) [39].

This study thus aimed at developing a French-Canadian scale that could be used to measure knowledge, beliefs, and attitudes that people in the community have towards CP. Our a priori hypothesis was that the CPMS would measure three constructs of knowledge, beliefs, and attitudes, that is, those towards people suffering from CP, biopsychosocial impacts of CP, and treatment of CP. The internal structure, internal consistency, and construct validity of the use of this scale named the Chronic Pain Myth Scale (CPMS) were also examined.

## 2. Methods

### 2.1. Development of the Chronic Pain Myth Scale

The CPMS was designed with the intent of providing a generic “one fits all” measurement tool that gives an overall index of the knowledge, beliefs, and attitudes that people have towards CP. It was developed through a multistep process according to a number of recommendations for the development of health measurement scales [41]. Preliminary items about knowledge, beliefs, and attitudes towards people suffering from CP, its biopsychosocial impacts, and its treatment were derived from different information sources. First, an email consultation was conducted among various key informants and pain experts, including 1 patient suffering from CP, 1 primary care nurse practitioner, 4 researchers in the field of pain, and 7 clinician-researchers in the field of pain (1 family physician, 2 anesthesiologists, 1 nurse, 1 dentist, 1 pharmacist, and 1 psychologist). These informants were chosen according to purposive sampling and asked to list negative beliefs and prejudices towards CP that are common in the community. Second, a comprehensive literature search was conducted to identify and review existing validated scales designed to measure knowledge, beliefs, attitudes, and perceptions about pain, that is, Back Beliefs Questionnaire (BBQ) [38, 39], Beliefs about Pain Control Questionnaire (BPCQ) [37], Health Care Providers' Pain and Impairment Relationship Scale (HC-PAIRS) [24], Knowledge and Attitudes Survey Regarding Pain (KASRP) [25], KnowPain-50 (KP-50) [23, 27], Pain and Impairment Relationship Scale (PAIRS) [36], Pain Attitudes and Beliefs Scale (PABS) [26, 29], Pain Beliefs and Perceptions Inventory (PBPI) [31, 32], Pain Beliefs Questionnaire (PBQ) [35], Pain Knowledge and Attitude (PAK) [28], Survey of Pain Attitudes (SOPA) [33, 34], and Toronto Pain Management Inventory (TPMI) [30]. Because most of these scales were originally designed for very specific populations (healthcare professionals, patients suffering from CP), many items had to be adapted for broader use in the community irrespective of the person's background. Finally, many educational and patient awareness websites, leaflets, and publications in mass media about CP were reviewed. Triangulation of these various data sources was achieved to develop a preliminary list of items as comprehensive as possible.

For the development of the CPMS, we avoided distinguishing between items measuring knowledge and those measuring attitudes, as suggested by the authors of the Knowledge and Attitudes Survey Regarding Pain (KASRP) [25]. A total of 51 preliminary items were formulated in statements that were then grouped into three categories depending on whether they were referring to knowledge, beliefs, and attitudes towards (1) people suffering from CP, (2) biopsychosocial impacts of CP, and (3) treatment of CP. Particular attention was paid to avoid unnecessary content duplication across items. Agreement with each statement was measured with a Likert scale, as it is the case in most of the tools (10/12) mentioned above. A 5-point Likert scale was chosen, ranging from 1 (completely disagree) to 5 (completely agree). The list of preliminary items was then reviewed by a convenience sample of 14 pain experts who attended the Annual Meeting of the Quebec Pain Research Network (http://www.qprn.ca/) (January 2014). These individuals received the paper-and-pencil questionnaire in their meeting package, including a cover letter asking them to annotate their suggestions about the clarity of the questionnaire, the formulation of the items, and the items to be removed/added. They were then invited to drop their responses at the front desk (14 out of 44 attendees provided their suggestions). Item reduction and reformulation were carried out by members of the study team based on the minor comments that were received (e.g., removal of few items, wording). The only major suggestion was to reverse the formulation of items (i.e., having to agree with some items and disagree with other items) in order to reduce acquiescence bias (yea-saying) [41, 42], which was done. The CPMS was then computerized on the web and pretested in a purposive sample of 4 individuals from the community having various socioeconomic status to ensure adequate comprehension of the items. The version of the CPMS to be validated had 44 items.

### 2.2. Validation Study Design and Settings

The validity of the use of the CPMS was evaluated in the context of a web-based cross-sectional study launched among the general population of the province of Quebec (Canada) between May and June 2014. Participants were eligible in this study if they were French-speaking Quebec residents, were aged 18 and older, and consented to complete the online questionnaire. Participants who did not complete all the items of the CPMS were excluded from the present validation study. The study was approved by the* Comité d'Éthique de la Recherche avec des Êtres Humains de l'Université du Québec en Abitibi-Témiscamingue* (February 21, 2014).

### 2.3. Procedure

The SurveyMonkey Gold® software (which allows for the direct transfer of survey data into IBM SPSS Statistics®) was used to put the study questionnaire online and collect data. The invitation to complete the questionnaire was disseminated using various diffusion platforms in order to reach a large number of individuals from all over the province and maximize our sample diversity and representativeness: (1) organizations that freely accepted to advertise the study invitation on their website, via their members' mailing list, or during their scheduled activities, (2) social media such as Facebook® and Twitter®, (3) paid publicity in provincial-wide mass media (ads in newspapers and e-papers), and (4) emails sent by the study team to research networks in the province of Quebec, colleagues, and contacts. The launching of this study also attracted media attention and was covered in numerous regional broadcasts and text interviews (Radio Canada®, RNC Media®, and Quebecor Media®). For all diffusion platforms, the invitation contained the questionnaire hyperlink (electronic invitations) or URL (paper ads). Potential participants were invited to go on the online introduction page of the study where enough information was provided to insure informed consent before the beginning of the questionnaire. The online questionnaire was available for a period of six weeks (May 1 and June 11, 2014).

In order to maximize participation rate, 10 prizes consisting of prepaid VISA® gift cards of Can$200 each were drawn from all participants who had completed the online questionnaire. A number of elements helped minimize double participation: (1) responses were limited to only one IP address, (2) responders were informed that only one participation per person was accepted, and (3) when it was time for participants to write at the end of the questionnaire their contact information in order to participate in the draw, those tempted to provide a false identity were discouraged by a statement explaining that prize winners will be asked to provide a copy of a valid piece of identification. Finally, careful attention was given to duplication of first and last name, telephone number, postal address, and email address during the database cleaning process.

In addition to the 44 items of the CPMS to be validated, the online questionnaire contained various items to assess the responders' sociodemographic characteristics (age, sex, country of birth, work status, education level, and region of residence). Participants were also asked if (1) they were suffering from pain every day or repeatedly for more than three months (i.e., considered as CP), if they knew someone who has CP, and (3) if they were a healthcare professional (i.e., physician, nurse, physiotherapist, psychologist, or pharmacist, the first four ones being identified by the International Association for the Study of Pain as clinicians who should ideally be implicated in multidisciplinary pain management) [43].

### 2.4. Data Analysis

Descriptive statistics were calculated to depict the participants' characteristics. Regarding CPMS's items, high values on positively worded items indicated better knowledge and more positive beliefs and attitudes towards people suffering from CP, biopsychosocial impacts of CP, and treatment of CP. Scores on negatively worded items were reversed so that a high value also indicated better perceptions. Using a principal axis factor extraction method (which is recommended when the data violate the assumption of multivariate normality) and an orthogonal Varimax rotation [44], exploratory factor analysis was used to explore the CPMS internal structure and to reduce the number of items for the final version. Calculation of the CPMS subscales derived from the exploratory factor analysis was conducted as follows. The scores of the three subscales of the CPMS were obtained by summing the items clustered during the exploratory factor analysis (see the appendix for the final version of the CPMS and calculation method). Unstandardized Cronbach's alpha coefficients (*α*) were used to assess the internal consistency of the three CPMS subscales. These coefficients measuring the intercorrelations between the items of a scale [45] range between 0 (weak reliability) and 1 (perfect reliability) and a cut-off ≥0.7 is commonly used as an indicator of adequate internal consistency/reliability for research purposes [46]. With regard to the construct validity of the use of a scale in a specific population, it can be determined by looking at whether scores are different in groups that should score significantly higher or lower on the construct that is supposed to be measured by the scale (known-groups or extreme groups technique) [41, 47]. Therefore, the CPMS subscales scores were compared between different subgroups: participants who reported suffering versus not suffering from CP, those who reported knowing versus not knowing someone who suffers from CP, and those who reported being versus not being a healthcare professional (Wilcoxon rank-sum tests). CPMS scores were also compared across sex groups. Finally, the relation between CPMS scores and participants' education level was assessed by calculating Spearman Correlation coefficients. All statistical analyses were performed using IBM SPSS Statistics version 22 and SAS® version 9.3.

## 3. Results

### 3.1. Participants' Characteristics

A total of 1958 participants from the 17 administrative regions of the province of Quebec completed the web-based questionnaire, among whom 1555 participants answered all the items of the CPMS. The proportion of participants who did not complete all the items of the CPMS was thus 20.6% and ranged between 16.7% and 24.2% across the predefined groups of participants (whether they had CP, knew someone who suffered from CP, or reported being a healthcare professional). Characteristics of the study sample are presented in Table 1. Participants' age varied from 18 to 83 years and 78.4% were women. A total of 69.6% reported suffering from CP, and 83.6% knew someone who was suffering from this condition. A minority of participants (14.5%) reported being a physician, a nurse, a physiotherapist, a psychologist, or a pharmacist.

### 3.2. Exploratory Factor Analysis and Item Reduction

**Figure 1:**
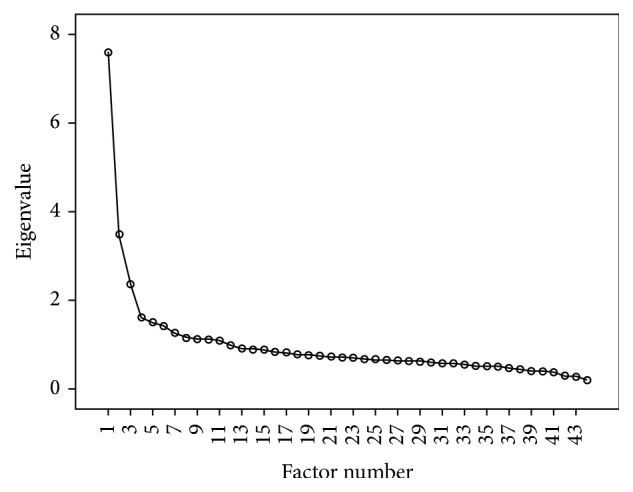
Scree plot.

The Kaiser-Meyer-Olkin measure underlined the sampling adequacy (KMO = 0.89) [48]. The initial factor analysis conducted among the 44 preliminary items of the scale revealed 11 different factors that had eigenvalues greater than Kaiser's criterion of 1 [48]. However, only 3 factors were retained based on the scree plot (see Figure 1) which can provide a reliable criterion for factor selection with a large sample size [48]. These three factors were also in accordance with our a priori hypothesis that the CPMS would measure three constructs of knowledge, beliefs, and attitudes, that is, those towards people suffering from CP, its biopsychosocial impacts, and its treatment. Based on the rotated factor matrix, items presenting loadings that rounded below 0.4 [48] on all three factors were removed from the analysis.  Table 2 shows the final factor loadings after rotation. The items that clustered on the same factors suggest that factor 1 best represents knowledge, beliefs, and attitudes towards people suffering from CP (9 items), factor 2 knowledge, beliefs, and attitudes towards biopsychosocial impacts of CP (10 items), and factor 3 knowledge, beliefs, and attitudes towards treatment of CP (7 items); 96.15% of the items showed no crossloading (item loads ≥0.32 on two or more factors [44]). The final version of the CPMS thus contained 26 items. Descriptive statistics and floor and ceiling effects for each of the CPMS subscales are presented in Table 2. Less than 16% of participants achieved the lowest or highest possible scores, respectively, which can be considered acceptable [49].

### 3.3. CPMS's Internal Consistency

Cronbach's *α* coefficients for each of the three CPMS subscales are shown at the bottom of Table 2 (*α* = 0.72–0.82). All the *α* values reached the 0.7 cut-off suggesting adequate reliability of the CPMS subscales. The same was true among participants suffering (*α* = 0.72–0.78) versus not suffering from CP (*α* = 0.72–0.85), those who reported knowing (*α* = 0.72–0.81) versus not knowing someone who suffers from CP (*α* = 0.69–0.83), and those who reported being (*α* = 0.73–0.79) versus not being a healthcare professional (*α* = 0.70–0.82).

### 3.4. CPMS's Construct Validity

**Figure 2:**
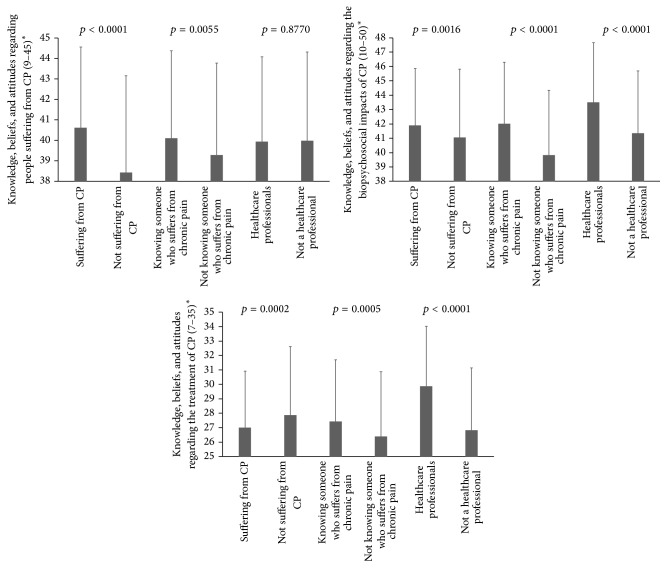
Comparison of CPMS subscales scores between different subgroups of participants. ^*∗*^Higher scores indicate higher knowledge and more positive beliefs and attitudes towards CP, its biopsychosocial impacts, and its treatment. The numbers in brackets refer to the range of possible scores for each of the three subscales. CPMS: Chronic Pain Myth Scale.

Comparisons of CPMS subscales scores between the predefined groups of participants are presented in Figure 2. Statistically significant group differences (*p* < 0.05) were found, the CPMS scores being higher on average among participants who reported suffering from CP (2/3 subscales), in those who knew someone who suffered from CP (3/3 subscales), and in those who were a healthcare professional (2/3 subscales). The only exceptions were regarding the first subscale (knowledge, beliefs, and attitudes regarding people suffering from CP), where no difference was found between participants reporting being versus not being a healthcare professional (*p* = 0.8770), and the third subscale (knowledge, beliefs, and attitudes regarding treatment of CP), where the score was higher among participants not suffering from CP (*p* = 0.0002). Differences were also found between women and men, with women presenting higher scores on the three CPMS subscales (people suffering from CP subscale: 40.22 ± 4.24 versus 38.94 ± 4.44; biopsychosocial impacts of CP subscale: 41.87 ± 4.77 versus 40.83 ± 5.00; treatment of CP subscale: 27.36 ± 4.25 versus 26.81 ± 4.36; all *p* < 0.05). Statistically significant correlations were also found between CPMS scores and the education level of participants (people suffering from CP subscale: *r* = −0.13; biopsychosocial impacts of CP subscale: *r* = 0.06; treatment of CP subscale: *r* = 0.25; all *p* < 0.05).

## 4. Discussion

Many scales are presently available to measure perceptions towards pain. However, none of these scales were specifically designed to be broadly used in the community whether the person suffers or not from CP or is a healthcare professional or not. This study thus aimed at developing such a type of scale to measure knowledge, beliefs, and attitudes people have towards persons having CP, the biopsychosocial impacts of this type of disorder, and its treatment. The results of our study, which was carried out in French-speaking individuals in the province of Quebec, provide preliminary insights into the psychometric qualities of the use of the CPMS in terms of internal structure, internal consistency, and construct validity.

Careful attention was given to the different steps of the CPMS development in order to maximize its content validity (i.e., the extent to which the scale measures all relevant content or domains of the topic it is supposed to measure [41]) and its internal structure. All estimated coefficients of internal consistency of the CPMS subscales showed adequate reliability (*α* > 0.7) which supports its use for research purposes in large populations [46]. Whether the participants were suffering from CP did not alter the CPMS reliability statistics.

Using the known-groups technique, preliminary arguments towards the construct validity of the use of the CPMS are also provided; that is, the majority of CPMS scores revealed better knowledge and more positive attitudes in groups that were expected to score higher on the scale such as patients having CP and participants who knew someone who suffered from CP. These results are consistent with validation studies of other scales used to survey knowledge, beliefs, and attitudes about pain which reported significantly different scores between pain patients and pain-free controls [35, 38, 40]. No difference regarding the knowledge, beliefs, and attitudes regarding people suffering from CP subscale was found between participants reporting being versus not being a healthcare professional. This result is not surprising considering that several studies reported on stigmatization of patients suffering from CP from healthcare professionals [20, 21].

CPMS scores were significantly higher among women (3/3 subscales) and associated with higher education levels (2/3 subscales), which also argue in favour of the construct validity of the scale. In fact, other studies have shown better knowledge and more positive attitudes regarding chronic diseases among female participants [26, 50] and more educated individuals [51, 52]. Effect sizes of bivariate subgroups analyses were small but could be explained by the fact that knowledge, beliefs, and attitudes are a multifactorial concept that cannot be explained by only one variable such as sex or education level. Although the approaches used in the present study are informative and necessary, future studies should however examine other aspects of the CPMS construct validity such as its ability to predict scores of other validated tools measuring related constructs.

Considering that the lack of recognition of CP is a barrier to its optimal management [16, 53], providing a new tool for the measurement of knowledge, beliefs, and attitudes towards CP in the community is most useful to better design and tailor awareness and education programs. That could ultimately improve CP prevention, recognition, and management. Besides the computation of CPMS total subscales scores, individual items of the scale could possibly be used separately to better describe specific knowledge, beliefs, and attitudes towards CP.

### 4.1. Strengths and Limitations

The present study has several strengths such as its large sample size (in particular for factor analysis that requires more than 5–20 times more respondents than the number of items to be analyzed [41]), in addition to the fact that participants had various socioeconomic profiles and came from different geographic areas of the province of Quebec, thus maximizing the external validity of our study. However, it should not be viewed as a population-based study given the characteristics of our sample (e.g., large proportion of responders suffering from CP, large proportion of women) which may have introduced a participation bias. In fact, during the study advertising, it was expected that patients suffering from CP or people who have an interest for pain would be more inclined to take part in our study. However, we managed to recruit many individuals that should be representative of the general public (i.e., neither those who were suffering from chronic pain nor healthcare professionals).

A 5-point Likert scale was chosen for the CPMS. However, odd-numbered scales present a neutral middle option (neither agree nor disagree) that could be misinterpreted by respondents (e.g., do not know). For this reason, we cannot exclude the possibility that the use of a different number of points on the Likert scale or even a different scale format could affect the results. We would note, however, that this issue did not come out during the expert review or the pretest of the CPMS. As for the reliability and validity of the CPMS, other studies should target the full range of psychometric properties (e.g., confirmatory factor analysis in different validation samples, test-retest reliability, convergent construct validity, sensitivity to change, and clinically important differences). Moreover, reliability and validity are not fixed psychometric properties of a scale and are unique to its use for a given patient population [41]. For this reason, we cannot assume that the psychometric properties assessed in our study will be exactly the same in other populations or in smaller studies.

## 5. Conclusion

Globally, the results of this study suggest that the CPMS could be a valuable general-purpose tool for research among French-speaking populations of the province of Quebec. This new scale could be useful to better describe knowledge, beliefs, and attitudes of the general public that could help to design successful awareness and education activities about the importance of CP, its impacts, and the ways to prevent it. Next steps would be to report on additional psychometric properties of the CPMS, which could eventually be used to help generate evidence supporting benefits of education programs, awareness campaigns, and stigma-reduction activities.

## Figures and Tables

**Table 1:** Study population's characteristics.

Characteristics^*∗*^ *n* = 1555	Number (%) of participants^*∗∗*^
*Age* (mean ± SD)	48.21 ± 13.11
Min	18
Max	83

*Sex*	
Females	1205 (78.40)
Males	332 (21.60)

*Country of birth *	
Canada	1434 (93.66)
Other	97 (6.34)

*Work status*	
Full-time job	772 (50.36)
Part-time job	127 (8.28)
Not working	634 (41.36)

*Completed education level*	
Elementary	15 (0.97)
High school	248 (16.11)
Diploma in vocational studies	201 (13.06)
College/CÉGEP	324 (21.05)
University, undergraduate studies	443 (28.78)
University, graduate studies	308 (20.01)

*Region* ^†^	
Nonremote regions	1081 (71.35)
Remote resource regions	434 (28.65)

*Suffering from CP (pain for ≥ 3 months)*	
Yes	1070 (69.62)
No	467 (30.38)

*Knowing someone who suffers from CP*	
Yes	1287 (83.57)
No	253 (16.43)

*Healthcare professionals* ^††^	
Yes	222 (14.52)
No	1307 (85.48)

^*∗*^Proportion of missing data ≤ 2.6%.

^*∗∗*^Unless stated otherwise.

^†^Remote resource regions as defined by *Revenu Québec* (i.e., the provincial revenue agency): *Bas-Saint-Laurent* (region 01), *Saguenay–Lac-Saint-Jean* (region 02), *Abitibi-Témiscamingue* (region 08), *Côte-Nord* (region 09), *Nord-du-Québec* (region 10), and *Gaspésie–Îles-de-la-Madeleine* (region 11). Nonremote regions are near major urban centres.

^††^Physician, nurse, physiotherapist, psychologist, or pharmacist.

CP: chronic pain; SD: standard deviation.

**Table 2:** Rotated factor matrix of the final version of the CPMS (*n* = 1555).

Unvalidated English translation of the 26 items of the French-Canadian CPMS^*∗*^	Factors		
	1	2	3
(1) *[Really have pain, it's not in their head]*	**,44**	,25	−,01
(2) *[Just want to be prescribed drugs]*	**,72**	,10	,03
(3) *[Seek sick leaves to stop working]*	**,84**	,06	,03
(4) *[Just want to laze and do not accomplish their daily tasks]*	**,83**	,14	,06
(5) *[Complain of pain to get attention from others]*	**,79**	,06	−,03
(6) *[Really want to get better]*	**,49**	,19	−,04
(7) *[Complain about their pain, but continue their activities (e.g., sports, snowmobile). Their pain should not be that bad]*	**,46**	,20	,06
(8) *[Become dependent to their medications such as drug addicts]*	**,35**	,02	,08
(9) *[Often tend to exaggerate the severity of their condition]*	**,62**	,14	,00
(10) *[Chronic pain causes several physical symptoms (e.g., muscle tension, change in appetite, reduced mobility, fatigue)]*	,33	**,46**	,11
(11) *[Chronic pain can have a direct impact on sexual life]*	,20	**,43**	,08
(12) *[People with chronic pain are sometimes rejected by their relatives]*	,09	**,55**	,14
(13) *[Chronic pain may be associated with negative emotions (e.g., fear, anger or sadness)]*	,14	**,49**	,08
(14) *[People with chronic pain tend not to isolate themselves]*	,23	**,43**	,13
(15) *[People with chronic pain usually have more difficulty to resist stressful events of everyday life]*	,08	**,42**	,06
(16) *[The risk of death by suicide is higher among people with chronic pain than in the general population]*	,08	**,52**	,03
(17) *[Chronic pain costs billions of dollars to our society]*	−,08	**,40**	,14
(18) *[People with chronic pain do not always have access to healthcare services to treat their condition]*	,16	**,44**	,09
(19) *[Doctors lack time to treat chronic pain]*.	,06	**,42**	,07
(20) *[Consulting a psychologist is useless unless the person with chronic pain is depressed]*	,07	,19	**,41**
(21) *[There is not much to do to improve chronic pain]*	,09	,11	**,42**
(22) *[Good sleeping habits help reduce chronic pain]*	−,10	,27	**,61**
(23) *[A balanced diet helps to reduce chronic pain]*	−,12	,22	**,60**
(24) *[Doing physical exercise may aggravate chronic pain]*	,08	,02	**,58**
(25) *[Working may aggravate chronic pain]*	,02	−,12	**,52**
(26) *[The treatment of chronic pain is in the hands of health care professionals and not those of the patient]*	,06	,26	**,42**

*Eigenvalues before rotation*	5.48	3.08	1.86

*Percentage of variance explained after rotation*	15.47	9.78	7.58

*Cronbach's α (items included in each factor)* ^*∗∗*^	0.82	0.74	0.72

*Descriptive statistics*			
Mean score ± SD	39.95 ± 4.32	41.64 ± 4.84	27.25 ± 4.28
Median (range)	41 (17–45)	42 (19–50)	27 (7–35)
Possible scores	9–45	10–50	7–35
% of respondents who achieved the lowest possible score	0.00%	0.00%	0.06%
% of respondents who achieved the highest possible score	15.50%	4.31%	4.50%

Bold type indicates primary factor loading for each item.

Factor 1: knowledge, beliefs, and attitudes towards people suffering from CP.

Factor 2: knowledge, beliefs, and attitudes towards biopsychosocial impacts of CP.

Factor 3: knowledge, beliefs, and attitudes towards treatment of CP.

CP: chronic pain; CPMS: Chronic Pain Myth Scale.

^*∗*^For publication purposes, an unvalidated English translation of the 26 items of the CPMS is presented. The validated French-Canadian version of the items can be found in the appendix.

^*∗∗*^Cronbach's *α* ≥ 0.7 = adequate internal consistency for research purposes.

## Appendix

### Échelle des Connaissances, Attitudes et Croyances de la Population envers la Douleur Chronique, Ses Impacts et Son Traitement

Les énoncés suivants portent sur votre perception des gens qui souffrent de douleur chronique.

Veuillez indiquer dans quelle mesure vous êtes d'accord avec ces énoncés sur une échelle allant de “Pas du tout d'accord” à “Tout à fait d'accord”.

Les gens qui souffrent de douleur chronique...

(1) Ont vraiment mal, ce n'est pas dans leur tête.
  □ Pas du tout d'accord
  □ Pas d'accord
  □ Ni d'accord, ni en désaccord
  □ D'accord
  □ Tout à fait d'accord
(2) Veulent juste se faire prescrire des médicaments.
  □ Pas du tout d'accord
  □ Pas d'accord
  □ Ni d'accord, ni en désaccord
  □ D'accord
  □∗ Tout à fait d'accord
(3) Cherchent à obtenir des congés de maladie pour ne plus travailler.
  □ Pas du tout d'accord
  □ Pas d'accord
  □ Ni d'accord, ni en désaccord
  □ D'accord
  □∗ Tout à fait d'accord
(4) Veulent juste paresser et ne pas accomplir leurs tâches quotidiennes.
  □ Pas du tout d'accord
  □ Pas d'accord
  □ Ni d'accord, ni en désaccord
  □ D'accord
  □∗ Tout à fait d'accord
(5) Se plaignent de douleurs pour obtenir de l'attention de la part des autres.
  □ Pas du tout d'accord
  □ Pas d'accord
  □ Ni d'accord, ni en désaccord
  □ D'accord
  □∗ Tout à fait d'accord
(6) Veulent vraiment aller mieux.
  □ Pas du tout d'accord
  □ Pas d'accord
  □ Ni d'accord, ni en désaccord
  □ D'accord
  □ Tout à fait d'accord
(7) Se plaignent de leur douleur, mais continuent leurs activités (p.ex., sports, motoneige). Leur douleur ne doit pas être si pire que ça.
  □ Pas du tout d'accord
  □ Pas d'accord
  □ Ni d'accord, ni en désaccord
  □ D'accord
  □∗ Tout à fait d'accord
(8) Deviennent dépendants à leurs médicaments (comme des toxicomanes).
  □ Pas du tout d'accord
  □ Pas d'accord
  □ Ni d'accord, ni en désaccord
  □ D'accord
  □∗ Tout à fait d'accord
(9) Ont souvent tendance à exagérer la sévérité de leur condition.
  □ Pas du tout d'accord
  □ Pas d'accord
  □ Ni d'accord, ni en désaccord
  □ D'accord
  □∗ Tout à fait d'accord

Les énoncés suivants portent sur votre perception des impacts de la douleur chronique.

Veuillez indiquer dans quelle mesure vous êtes d'accord avec ces énoncés sur une échelle allant de “Pas du tout d'accord” à “Tout à fait d'accord”.

(10) La douleur chronique provoque plusieurs symptômes physiques (p.ex., tensions musculaires, modification de l'appétit, réduction de la mobilité, fatigue).
  □ Pas du tout d'accord
  □ Pas d'accord
  □ Ni d'accord, ni en désaccord
  □ D'accord
  □ Tout à fait d'accord
(11) La douleur chronique peut avoir un effet direct sur la vie sexuelle.
  □ Pas du tout d'accord
  □ Pas d'accord
  □ Ni d'accord, ni en désaccord
  □ D'accord
  □ Tout à fait d'accord
(12) Les gens souffrant de douleur chronique sont parfois rejetés par leurs proches.
  □ Pas du tout d'accord
  □ Pas d'accord
  □ Ni d'accord, ni en désaccord
  □ D'accord
  □ Tout à fait d'accord
(13) La douleur chronique peut s'accompagner d'émotions négatives (p.ex., peur, colère ou tristesse).
  □ Pas du tout d'accord
  □ Pas d'accord
  □ Ni d'accord, ni en désaccord
  □ D'accord
  □ Tout à fait d'accord
(14) Les gens souffrant de douleur chronique n'ont pas tendance à s'isoler.
  □ Pas du tout d'accord
  □ Pas d'accord
  □ Ni d'accord, ni en désaccord
  □ D'accord
  □∗ Tout à fait d'accord
(15) Les gens souffrant de douleur chronique ont généralement plus de difficulté à résister aux événements stressants de la vie quotidienne.
  □ Pas du tout d'accord
  □ Pas d'accord
  □ Ni d'accord, ni en désaccord
  □ D'accord
  □ Tout à fait d'accord
(16) Les risques de décès par suicide sont plus élevés chez les gens atteints de douleur chronique que dans la population générale.
  □ Pas du tout d'accord
  □ Pas d'accord
  □ Ni d'accord, ni en désaccord
  □ D'accord
  □ Tout à fait d'accord
(17) La douleur chronique coûte des milliards de dollars à notre société.
  □ Pas du tout d'accord
  □ Pas d'accord
  □ Ni d'accord, ni en désaccord
  □ D'accord
  □ Tout à fait d'accord
(18) Les gens souffrant de douleur chronique n'ont pas toujours accès aux services de santé pour les soigner.
  □ Pas du tout d'accord
  □ Pas d'accord
  □ Ni d'accord, ni en désaccord
  □ D'accord
  □ Tout à fait d'accord
(19) Les médecins manquent de temps pour traiter les cas de douleur chronique.
  □ Pas du tout d'accord
  □ Pas d'accord
  □ Ni d'accord, ni en désaccord
  □ D'accord
  □ Tout à fait d'accord

Les énoncés suivants portent sur votre perception des traitements pour la douleur chronique.

Veuillez indiquer dans quelle mesure vous êtes d'accord avec ces énoncés sur une échelle allant de “Pas du tout d'accord” à “Tout à fait d'accord”.

(20) Consulter un psychologue ne sert à rien, sauf si la personne souffrant de douleur chronique est déprimée.
  □ Pas du tout d'accord
  □ Pas d'accord
  □ Ni d'accord, ni en désaccord
  □ D'accord
  □∗ Tout à fait d'accord
(21) Il n'y a pas grand-chose à faire pour améliorer la douleur chronique.
  □ Pas du tout d'accord
  □ Pas d'accord
  □ Ni d'accord, ni en désaccord
  □ D'accord
  □∗ Tout à fait d'accord
(22) De bonnes habitudes de sommeil contribuent à réduire la douleur chronique.
  □ Pas du tout d'accord
  □ Pas d'accord
  □ Ni d'accord, ni en désaccord
  □ D'accord
  □ Tout à fait d'accord
(23) Une alimentation équilibrée contribue à réduire la douleur chronique.
  □ Pas du tout d'accord
  □ Pas d'accord
  □ Ni d'accord, ni en désaccord
  □ D'accord
  □ Tout à fait d'accord
(24) Faire de l'exercice physique risque d'aggraver la douleur chronique.
  □ Pas du tout d'accord
  □ Pas d'accord
  □ Ni d'accord, ni en désaccord
  □ D'accord
  □∗ Tout à fait d'accord
(25) Travailler risque d'aggraver la douleur chronique.
  □ Pas du tout d'accord
  □ Pas d'accord
  □ Ni d'accord, ni en désaccord
  □ D'accord
  □∗ Tout à fait d'accord
(26) Le traitement de la douleur chronique c'est entre les mains des professionnels de la santé et non celles du patient.
  □ Pas du tout d'accord
  □ Pas d'accord
  □ Ni d'accord, ni en désaccord
  □ D'accord
  □∗ Tout à fait d'accord

*Calcul des Scores*. Les scores sont obtenus en effectuant la somme des items propres à chacune des dimensions:

1. Connaissances, attitudes et croyances en regard des personnes qui souffrent de douleur chronique (somme des items (1) à (9)).
2. Connaissances, attitudes et croyances en regard des impacts de biopsychosociaux de la douleur chronique (somme des items (10) à (19)).
3. Connaissances, attitudes et croyances en regard du traitement de la douleur chronique (somme des items (20) à (26)).

Accorder des points s'étendant de 1 pour la réponse “Pas du tout d'accord” à 5 pour la réponse “Tout à fait d'accord”.

Pour les items formulés négativement^*∗*^, inverser les scores lors des analyses afin qu'un score total plus élevé corresponde à une meilleure connaissance ainsi que des attitudes et croyances plus positives envers la douleur chronique, ses impacts et son traitement.

## Additional Points

*Summary*. This study aimed at developing and validating the Chronic Pain Myth Scale (CPMS), a French-Canadian scale for the measurement of knowledge, beliefs, and attitudes of people in the community towards individuals suffering from chronic pain (CP), biopsychosocial impacts of CP, and treatment of CP. The results of this web-based cross-sectional study conducted among the Quebec population provide preliminary evidence suggesting that the CPMS could be a valuable tool for research among French-speaking populations.
